# Supplementary material for: Retinoids as Alternative Antifungal Agents Against Candida albicans: In Vitro and In Silico Evidence
Source: Microorganisms. 2025 Jan 22;13(2):237. doi: 10.3390/microorganisms13020237 (PMC11857849; doi:10.3390/microorganisms13020237)
Supplement: Supplementary file 1 [file microorganisms-13-00237-s001.zip › microorganisms-3405571-supplementary.pdf]

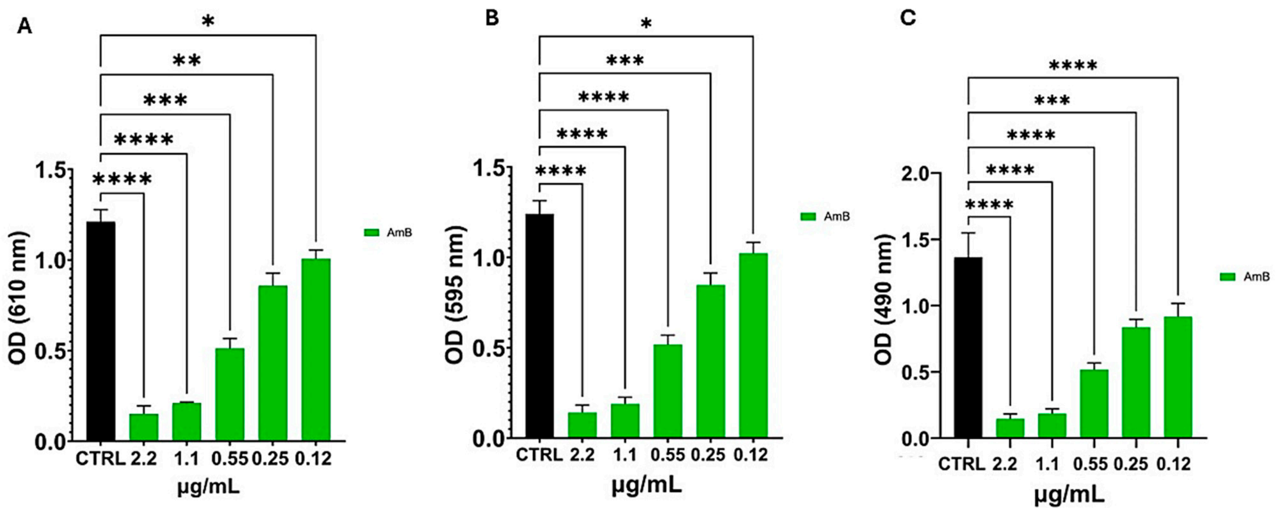

Figure S1. AmB (2–0.12 µg/mL) was used as positive control drug for *C. albicans* growth (A), biofilm biomass (B) and metabolic activity (C). The absorbance intensity of the crystal violet dye was measured using a spectrophotometer plate reader at 610, 595 and 490 nm, respectively. Results are the means ± SD of three independent experiments carried out in triplicate. One-way ANOVA, **p*<0.05; ** *p*<0.01; *** *p*<0.001; *****p*< 0.0001. AmB, Amphotericin B.
